# Strengthening causes of death identification through community-based verbal autopsy during the COVID-19 pandemic

**DOI:** 10.1186/s12889-022-14014-x

**Published:** 2022-08-23

**Authors:** Kemal Nazarudin Siregar, Rico Kurniawan, Dion Zein Nuridzin, Ryza Jazid BaharuddinNur, Retnowati, Yolanda Handayani, Rohjayanti, Lindawati Halim

**Affiliations:** 1 Department of Biostatistics and Population Studies, Faculty of Public Health, Universitas Indonesia, Depok City, Indonesia; 2 Health Informatics Research Cluster (HIRC) Faculty of Public Health, Universitas Indonesia, Depok City, Indonesia; 3 Department of Biostatistics and Population Studies, Faculty of Public Health, Universitas Diponegoro, Semarang City, Indonesia; 4 Center of Biostatistics and Health Informatics, Faculty of Public Health, Universitas Indonesia, Depok City, Indonesia; 5 Department Epidemiology, Faculty of Public Health, Universitas Hasanuddin, Makassar City, Indonesia; 6 Medical Record and Health Information Department, Semarang Health Polytechnic Ministry of Health, Semarang City, Indonesia; 7 Head of NCD Prevention and Control Section, Bogor District Health Office, Bogor District, Indonesia; 8 Health Center Coordinator in Babakan Madang Sub-District, Bogor District, Indonesia

**Keywords:** Verbal autopsy, Community-based verbal autopsy, Mobile-based verbal autopsy, Causes of death, Covid-19

## Abstract

**Introduction:**

Indonesia has not optimally provided complete and reliable civil registration and vital statistics (CRVS). Death certification is one of the elements of the CRVS system. Reliable data on death rates and causes serve as the basis for building a strong evidence base for public health policy, planning, monitoring, and evaluation. This study aims to implement an approach to identifying the cause of death through verbal autopsy by empowering community health workers during the pandemic.

**Method:**

This study is implementation research with the empowerment of the community, in this case, health cadres and health facilitators/workers, to identify the cause of death through a mobile-based verbal autopsy. This implementation research consisted of four main activities: community-based verbal autopsy, mobile-based verbal autopsy development, data collection, and analysis of the suspected causes of death using InterVA-5.

**Result:**

From October to November 2020, a total of 143 respondents were willing to do a verbal autopsy interview (response rate of 58%). Of 143 respondents, most of them were women (112 or 78.3%), was the child of the deceased (61 or 42.7%) and lived with the deceased until before he/she died (120 or 83.9%). Based on the characteristics of the deceased, of 143 deceased, 78 (54.5%) were male, 134 (93.7%) were adults, 100 (69.9%) died at home, and 119 (83.2%) did not have a death certificate stating the cause of death. The cause of death of 143 deceased mainly was infectious disease (92 or 64.3%), followed by non-communicable disease (39 or 27.3%), external factors (5 or 3.5%), and unknown factors (4 or 2.8%). In sequence, the top five suspected causes of death are acute respiratory infection, including pneumonia (72 or 50.3%), other and unspecified infectious disease (18 or 12.6%), other and unspecified cardiac disease (17 or 11.9%), acute cardiac disease (4 or 2.8%), and Digestive neoplasms (4 or 2.8%).

**Conclusion:**

The findings showed that the mobile-based verbal autopsy using a community-based mechanism was feasible during the COVID-19 pandemic.

## Introduction

Indonesia has not optimally provided complete and reliable civil registration and vital statistics (CRVS) from Department of Population and Civil Registration (*Dinas Kependudukan dan Pencatatan Sipil/Disdukcapil*) at the district level under The Ministry of Home Affairs (MoHA) [1]. Vital statistics depend on a country's legal framework but typically include birth, death, cause of death, marriage and divorce, as well as adoption [2].

Death certification is one of the elements of the CRVS system. In Indonesia, the death certification system in Indonesia is still weak, and the cause of death of people mostly remains unknown [3, 4]. Reliable data on the rates and causes of death serve as the basis for building a strong evidence base for public health policy, planning, monitoring, and evaluation [5, 6]. Accurate, real-time statistics on causes of death are critical to developing public health policies and supporting a country’s ability to respond to emerging health threats and epidemics [7]. The availability of robust data related to vital statistics, including causes of death, is important for calculating, accountability, and monitoring the progress of achieving the Sustainable Development Goals (SDGs) and national targets [5, 8–10].

Globally, two-thirds (38 million) of 56 million annual deaths are still not registered [11]. A comparison of the findings of the leading causes of death is influenced by variations in certification skills of physicians, availability of diagnostic and pathological data when filling out death certificates, differences in medical culture when selecting the root cause, and the availability of trained mortality coders to accurately code the cause of death information [12–14]. For other deaths without medical certification, multiple data sources and diagnostic approaches, including surveillance systems, demographic research sites, surveys, censuses, disease records, verbal autopsies, and police records, should be utilized to obtain an overview of the causes of death in various populations [15, 16].

Civil registration in Indonesia has not realized the usefulness of death information as a reliable source of data [17]. The mortality measurement is the basic indicator for evaluating population health and is best derived from the death registration system. Nonetheless, such information is unavailable in Indonesia [18]. Consequently, indirect demographic techniques and life table models were utilized to estimate Indonesian mortality [19, 20].

The best alternative without a death certificate to ascertain the probable cause of death is a verbal autopsy (VA). VA mostly discusses the causes of death of individuals in countries with inadequate civil registration and death certification systems, where the majority of people die at home without interacting with the health system. Previous research has shown that VA can improve the diagnosis of the cause of death, and this method continues to be used in many countries [21, 22]. The face-to-face interview has been the standard method of communication for VA interviews [23]. During the coronavirus disease 2019 (COVID-19) pandemic, the face-to-face VA process has been delayed due to the physical distancing preventive measures implemented [24]. During pandemics or other instances where face-to-face interviews are not possible, the telephone interview method ensures VA data collection is not delayed and provides accuracy for mortality data [25].

This study aims to implement an approach to identifying the cause of death through verbal autopsy by empowering community health workers during the pandemic. With this approach, it is hoped that quality and sustainable data and information on causes of death at the community level can be provided to strengthen the death registration system and improve health program planning.

## Method

This study is implementation research [26] with the empowerment of the community, in this case, health cadres and health facilitators/workers, to identify the cause of death through a mobile-based verbal autopsy. This study was conducted in Babakan Madang Sub-District, Bogor District, between September and December 2020. This implementation research consisted of four main activities, namely:

### Community-based verbal autopsy

This study developed a mechanism for empowering health cadres and health facilitators/workers in conducting a verbal autopsy to determine the suspected cause of death in the community. Health cadres were recruited by program managers at the public health centers based on their activeness in implementing health programs in their respective villages, while the researchers recruited health facilitators/workers. The health facilitators/workers involved in this study were specifically recruited for Verbal Autopsy, while the health cadres involved in this study were not specific for Verbal Autopsy but also carried out their routine work as health cadres. Then, the selected health cadres and facilitators/workers were given education, training, and role play related to the strategy for implementing verbal autopsy. A total of 18 health cadres were selected from nine villages, and nine health facilitators/workers participated in the training.

### Mobile-based verbal autopsy

This mobile-based verbal autopsy application was developed using the Kobotoolbox platform with the questions adopted based on the WHO 2016 verbal autopsy instrument version 1.5.3 and translated to Indonesian [27]. All the input data were stored in the cloud server of the data collection application using the Kobo toolbox platform.

### Data collection

This research involved the residents of Babakan Madang Sub-District aged 18 years and over, who had WhatsApp, and family members who died within six weeks to 12 months before the study. The selection of the period between the date of death and the interview date was based on the VA Field Interviewer Manual for 2016 WHO VA Instrument [28]. The minimum period of six weeks allows sufficient time for the family to recover from the bereavement and be emotionally ready to share details of the event, while the maximal period of 12 months has been designed to reduce the potential for details to be forgotten or confused. Health cadres first recorded the people who died during that period and approached the family to perform a verbal autopsy. After they were ready, health facilitators/workers performed a verbal autopsy over the WhatsApp call (according to the health protocol during the COVID-19 pandemic) using a mobile-based verbal autopsy.

### Analysis of suspected causes of death

The collected verbal autopsy data were then adjusted to the InterVA-5 format (comma-separated values or.csv file) using Ms. Excel [29]*.* The formatted data were then analyzed using InterVA-5 to generate suspected causes of death for the individual. The InterVA-5 analysis generated up to three suspected specific causes of death with likelihood and provided an ICD-10 code according to the specific cause of death [27, 30]. The results of the suspected cause of death were reported in the aggregate at the sub-district level.

## Result

**Fig. 1:**
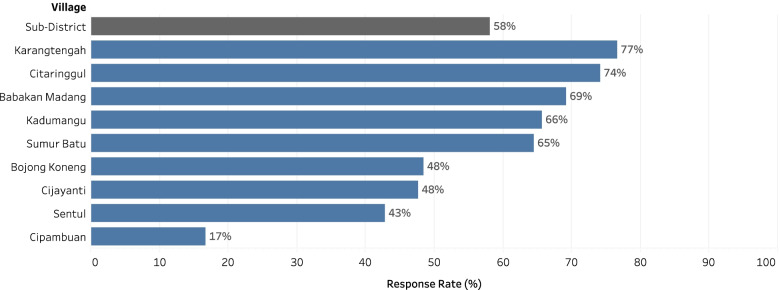
VA Response Rate in Babakan Madang Sub-District

During this COVID-19 pandemic, all activities are carried out online following applicable health protocols. Based on the data collected by cadres from local villages, it was identified that 246 people died from six weeks to 12 months before the study. Overall, from October to November 2020, 246 potential respondents were contacted by health cadres, and 143 respondents were willing for a verbal autopsy interview (response rate of 58%) (Fig. 1). Of the nine villages, five villages (Sumur Batu, Kadumangu, Babakan Madang, Citaringgul, and Karangtengah) had a response rate of more than 60%, and four villages (Bojong Koneng, Cijayanti, Sentul, and Cipambuan) had a response rate below 50%. Monitoring was carried out every week through weekly reports by health cadres and facilitators to ensure the quality of research activities. The active role of health cadres as local contacts is important to explain and approach family members so that health workers/facilitators can perform a verbal autopsy.

### Analysis of suspected causes of death

The characteristics of respondents who were willing to do a verbal autopsy are shown in Table 1. Of 143 respondents, most of them were women (112 or 78.3%), was the child of the deceased (61 or 42.7%) and lived with the deceased until before he/she died (120 or 83.9%). Based on the characteristics of the deceased, of 143 deceased, 78 (54.5%) were male, 134 (93.7%) were adults, 100 (69.9%) died at home, and 119 (83.2%) did not have a death certificate stating the cause of death.

**Table 1:** Characteristics of Verbal Autopsy Respondents in Babakan Madang Sub-District

Characteristics	n	%
**Characteristics of Interviewee**		
**Sex**		
Female	112	78.3
Male	31	21.7
**Relationship with the deceased**		
Child	61	42.7
Other family members	46	32.2
Spouse	22	15.4
Parent	14	9.8
Other relationships	1	0.7
**Living together with the deceased until before death**		
Yes	120	83.9
No	23	16.1
**Characteristics of the deceased**		
**Sex**		
Male	78	54.5
Female	65	45.5
**Age group**		
Adult (12 years and over)	134	93.7
Child (28 days to 11 years)	6	4.2
Newborn (0 to 27 days)	3	2.1
**Place of death**		
Home	100	69.9
Hospital	37	25.9
On the way to the hospital or health facility	4	2.8
Others	2	1.4
**Have a Death Certificate stating the cause of death**		
No	119	83.2
Yes	24	16.8
**Age at death (years) (Adult only, *n = *134)**		
15–24	8	6.0
25–34	3	2.2
35–44	14	10.4
45–54	16	11.9
55–64	40	29.9
65–74	25	18.7
75–84	17	12.7
85 +	11	8.2
**Marital Status (Adult only, *n = *134)**		
Married	83	61.9
Widow/widower	40	29.9
Unmarried	9	6.7
Divorced	2	1.5
**Highest education (Adult only, *n = *134)**		
Higher than middle school	16	11.9
Middle school/equivalent	10	7.5
Elementary school/equivalent	60	44.8
No formal education	39	29.1
Unknown	9	6.7
**Activity 1 year before death (Adult only, *n = *134)**		
Employee	69	51.5
Housewife	38	28.4
Unemployed	16	11.9
Retired	9	6.7
Student	2	1.5

Table 2 describes VA's suspected causes of death in Babakan Madang Sub-District and integration with ICD-10. The results showed that the cause of death of 143 deceased mainly was infectious disease (92 or 64.3%), followed by non-communicable disease (39 or 27.3%), external factors (5 or 3.5%), and unknown factors (4 or 2.8%). In sequence, the top five suspected causes of death are acute respiratory infection, including pneumonia (72 or 50.3%), other and unspecified infectious disease (18 or 12.6%), other and unspecified cardiac disease (17 or 11.9%), acute cardiac disease (4 or 2.8%), and Digestive neoplasms (4 or 2.8%).

**Table 2:** Suspected Causes of Death of Verbal Autopsy in Babakan Madang Sub-District

WHO VA cause of death category	ICD Code	n (143)	%
**Communicable Diseases**		**92**	**64.3**
01.02 Acute respiratory infection, including pneumonia	J00-J22	72	50.3
01.99 Other and unspecified infectious disease	A17-A19; A20-A38; A42-A89; B00- B19; B25-B49; B55-B99	17	11.9
10.03 Neonatal pneumonia	P23-P25	2	1.4
01.07 Meningitis and encephalitis	A39; G00-G05	1	0.7
**Non-Communicable Diseases**		**39**	**27.3**
04.99 Other and unspecified cardiac disease	I00-I09; I10-I15; I26-I52; I70-I99	18	12.6
04.01 Acute cardiac disease	I20-I25	4	2.8
02.05 & 02.06 Reproductive neoplasms MF	C51-C58 (Female); C60-C63 (Male)	3	2.1
02.02 Digestive neoplasms	C15-C26	4	2.8
06.01 Acute abdomen	R10	3	2.1
04.02 Stroke	I60-I69	2	1.4
**External Factor**		**5**	**3.5**
12.03 Accidental fall	W00-W19	3	2.1
12.01 Road traffic accident	V01-V89	2	1.4
**Unknown**		**4**	**2.8**
99 Cause of death unknown	R95-R99	4	2.8

## Discussion

### The extent to which this system can identify suspected causes of death at the community level

Currently, the government lacks information regarding the specific causes of death at the community level. Knowing the cause of death at the population/community level is crucial since significant disparities exist between the causes of death in the community and the medical setting [31]. Based on data from Bogor District Central Statistic Agency in 2018, the number of deaths in Babakan Madang Sub-District was 702 deaths [32]. However, only 31 death certificates (4.4%) were issued by the Population and Civil Registration Service [32, 33]. The low percentage of ownership of death certificates can occur because most deaths are outside health facilities and are not officially recorded in the passive surveillance system [34]. The results of the Sample Registration System also show that 64.5% of deaths in Indonesia occur at home [3]. The critical finding also showed that most deaths occurred outside health facilities (*n = *106, 74.1%).

The rules regarding reporting of deaths already exist, but they are still passive and rely on the family to report the incident of death to the head of the neighborhood unit for further reporting to Department of Population and Civil Registration no later than 30 days from the date of death [35]. There is also already a mechanism for reporting death data in health care facilities, in which every health care facility operator must report data on deaths and causes of natural and unnatural deaths to the local Health Office once a month, with a copy submitted to Department of Population and Civil Registration [35]. However, the data sharing mechanism between the Health Office and Department of Population and Civil Registration does not run optimally [36], resulting in a low death notification rate in the population. Thus, verbal autopsies using the WHO VA instrument are recommended.

The WHO VA instrument has been utilized extensively, particularly in low- and middle-income countries where routine data on causes of death are lacking and where most persons die at home [21, 22, 34, 37]. The analysis using InterVA-5 carried out in this study is also recommended by WHO as a tool for analyzing VA [27]. Previous research has shown that the results of VA analysis using InterVA-5 compared with established causes at participating tertiary hospitals can obtain concordance correlation coefficients of 0.92 for children and 0.86 for adults and provide the ICD-10 code according to the specific cause of death [30].

The results of this study indicate that the top five suspected causes of death are acute respiratory infection, including pneumonia, other and unspecified infectious disease, other and unspecified cardiac disease, acute cardiac disease, and pulmonary tuberculosis. In accordance with Wahab's research, the major cause of mortality is stroke (20.6%), followed by acute respiratory infections (ARIs), including pneumonia (15.7%), other and nonspecific cardiovascular illnesses (9.8%), malaria (6.0%), and tuberculosis (5.9%) [38]. According to national data, stroke is the main cause of death, followed by ischemic heart disease, diabetes, cirrhosis, and tuberculosis [39].﻿ There will be discrepancies when compared to national data on death causes. The result of this research is a pattern of causes of death that is representative of a limited community in the Babakan Madang subdistrict and occurs during a pandemic. However, the use of mobile-based verbal autopsies in this study seems to indicate that this approach has the potential to get data on causes of death at the population level, whereas these data are difficult to collect at the district level. Of course, further research in the broader area is needed to get a more solid conclusion.

### To what extent can this system be implemented during the COVID-19 pandemic

The COVID-19 pandemic has limited the mobility and face-to-face activities of the community-based verbal autopsy in this study, but with the use of information and communication technology, it can still be implemented. According to research from Siregar et al., in the conditions of the COVID-19 pandemic, community empowerment using information and communication technology has a high scope in reaching the community [40].

The mechanism developed in this study allows researchers, health service officers, public health center officers, health facilitators/workers, health cadres, and the community to be connected by using smartphones and communication media such as WhatsApp or Zoom. Previous research has shown that the use of smartphones is effective in various social learning and communication contexts in health and healthcare, including patient care, monitoring, rehabilitation, communication, diagnosis, education, and research [41]. Research conducted by Henry et al. showed that WhatsApp had exhibited a sort of innovation that can aid community-based initiatives in times of emergency [42]. Mandy et al. also stated that the use of the Zoom application as a tool for qualitative data collection has great potential due to its relative ease of use, cost-effectiveness, data management features, and security options [43]. A study from Nasarudin et al. also said that VA via telephone interviews was feasible, acceptable, and could be used as an alternative to face-to-face interviews without affecting the data quality. As long as face-to-face interviews are not possible, VA telephone interviews can be used for data collection for mortality surveillance [25].

All activities in this study, ranging from meetings, training, implementation, and monitoring to evaluation, were carried out online via Zoom Meetings or WhatsApp calls. Although the response rate in the four regions was below 50% and several obstacles were found, this activity could be carried out well during the pandemic. After the pandemic, of course, this research needs to be continued so that the effectiveness of face-to-face in this community-based verbal autopsy can also be studied.

There are some obstacles and challenges in community-based VA. First, the prospective respondents refused and were offended because they were asked about their deceased family members. There was still a sense of loss, which made them sad, and some people believe that asking about the deceased is not permissible by religion. Therefore, health cadres were asked to explain the objectives of the VA research according to the format provided, in a kind, polite, and personal way to build trust, but if they still refused, the health cadres could contact other prospective respondents. Second, the respondents feared their data being misused as the data of COVID-19 patients. The health cadres were asked to explain that the respondents’ data would not be misused and that this activity had received approval and guidance from the Bogor District Health Office and the local public health center. Third, as a mode of communication in implementing VA in the field, Whatsapp use has challenges related to connectivity experienced by health cadres and respondents. For example, limited internet quota, lack of digital literacy, and the existence of remote areas in several villages so that access to telephone or internet networks is inadequate.

### Potential for integrating with health and demographic surveillance system and routine data

In 2019, the researchers conducted a pilot study of the Health and Demographic Surveillance System (HDSS) in the Babakan Madang Sub-District, which was planned to be carried out continuously. The application of HDSS in Babakan Madang Sub-District could provide vital statistics, including births, deaths, transfers (in and out), and health situations through PIS-PK (*Program Indonesia Sehat-Pendekatan Keluarga*/Healthy Indonesia Program through Family Approach) which a passive surveillance system cannot record. With the HDSS in Babakan Madang Sub-District, data on the deceased have been collected, but the cause is not yet known. Integration with existing systems such as routine data recording in health facilities, vital statistics, and civil registration is also required to link death-related registration. Integrating death data and the implementation of mobile-based VA to find out the suspected causes of death in the population allows policymakers to know Cause-Specific Death rates at the population level. Several studies have also shown the possible integration between VA and HDSS to corroborate vital statistics and cause-specific mortality figures at the population level [44–46]. This integration can strengthen local potentials that ensure the functioning of health cadres to be actively involved in participatory community-based health programs. Additionally, prior research has emphasized the need for a functional community surveillance system to help record accountable deaths [40].

### Limitation

The level of acceptance of families to be interviewed regarding the death of their family members can vary, so the activeness of health cadres is very important in reaching and explaining to them. In addition, there are remote areas in this sub-district with no internet network, so using WhatsApp calls/Zoom Meetings is not possible. Thus, the deaths in the work area of ​​each cadre cannot be recorded.

We did not validate and confirm the cause of death with physicians. Moreover, most of the deaths occurred at home, so no historical data were found for analyzing the causes of death in health services. This can lead to misclassification of the suspected causes of death or unspecified suspected death in some individuals. With the VA method, which in the future could be integrated with existing systems such as routine data recording in health facilities, vital statistics, and civil registration, it is hoped that program holders and policymakers can obtain an unprecedented overview of the causes of death at the population level.

## Conclusion

The findings indicated that VA using a community-based mechanism was feasible to run during the COVID-19 pandemic. The mobile-based VA application can also be used to identify the causes of death at the population level. The availability of data related to suspected causes of death at the population level can hopefully serve as the basis for health program holders in making policies and plans to improve public health.

### Recommendation

Integrating death registration with HDSS and existing systems such as routine data recorded in health facilities, vital statistics, and civil registration is needed to strengthen death registration and implement VA. For some areas that lack internet access, if face-to-face verbal autopsies are still not possible, VA can be done by telephone (no internet signal required).

## Data Availability

The datasets generated and analyzed during the current study are not publicly available due to recommendations from the ethics commission but are available from the corresponding author on reasonable request.

## Abbreviations

COVID-19: Coronavirus Disease-19
CRVS: Civil Registration and Vital Statistics
CSV: Comma-separated values
Disdukcapil: Dinas Kependudukan dan Pencatatan Sipil (Department of Population and Civil Registration)
HDSS: Health and Demographic Surveillance System
ICD: International Classification of Diseases
MoHA: Ministry of Home Affairs
PIS-PK: Program Indonesia Sehat dengan Pendekatan Keluarga (Healthy Indonesia Program through Family Approach)
SDGs: Sustainable Development Goals
VA: Verbal Autopsy
WA: WhatsApp
WHO: World Health Organization
